# Perceived neighborhood safety, crime exposure, and chronic diseases among older Indians: The role of functional disabilities

**DOI:** 10.1371/journal.pgph.0005151

**Published:** 2025-09-24

**Authors:** Manacy Pai, T. Muhammad, Adedayo Adeagbo, Waad Ali

**Affiliations:** 1 Department of Sociology and Criminology, Kent State University, Kent, Ohio, United States of America; 2 Department of Human Health and Family Studies, Pennsylvania State University, University Park, Pennsylvania, United States of America; 3 Department of Sociology and Criminology, Kent State University, Kent, Ohio, United States of America; 4 Department of Geography, Sultan Qaboos University, Muscat, Oman; PLOS: Public Library of Science, UNITED STATES OF AMERICA

## Abstract

We examined the associations between perceived neighborhood safety, crime exposure, and the prevalence of chronic conditions and multimorbidity among older adults in India. Moreover, we examined whether these associations varied by functional disabilities measured by difficulties in activities of daily living (ADLs) and instrumental activities of daily living (IADLs). Data came from the World Health Organization’s Study on Global AGEing and Adult Health (SAGE), India Wave 2, conducted in 2015. Neighborhood safety was measured by perceptions of safety at home and while walking after dark, whereas crime exposure was assessed through reports of household victimization by violent crime in the past year. Chronic conditions were self-reported physician diagnoses of hypertension, diabetes, stroke, arthritis, angina, asthma, and lung disease, with multimorbidity defined as the presence of more than two chronic diseases. Multivariable regression analyses were used to examine the main associations and interaction terms to test the moderating role of ADL/IADL disabilities. The mean score of neighborhood safety (on a scale of 0–10) was 7.72 (SD: 2.05). Approximately 6% of the participants reported that they or someone in their household had been victims of violent crime in the previous year. Older adults with perceived neighborhood safety reported a lower number of chronic conditions (adjusted beta: -0.03, confidence interval [CI]: -0.04 to 0.01) and lower odds of multimorbidity (adjusted OR: 0.95, CI: 0.91 – 0.99). Those with crime exposure reported a higher number of chronic conditions (adjusted beta: 0.10, CI: 0.02 – 0.19). These associations were significantly more pronounced among those with ADL/IADL disabilities. Perceived neighborhood safety and crime exposure were significantly linked to chronic diseases and multimorbidity among older adults in India, particularly among those with functional disabilities. These findings underscore the need for targeted strategies to improve neighborhood safety and support among older Indians with functional disabilities.

## Background

A longer life expectancy means that people live longer than before. However, the gap between life expectancy and healthy life expectancy, particularly in low- and middle-income countries (LMICs), is alarming [1]. While older adults in LMICs live longer, they endure a rising burden of chronic conditions and multimorbidity [2–4], with nearly 80% of premature mortality from chronic diseases occurring in these countries [5,6]. To facilitate healthy aging, it is important to address various factors that contribute to the prevalence of chronic conditions among older adults. Among these, neighborhood conditions stand out as a significant determinant of health and well-being [7–9]. The environment in which older adults live shapes their physical, mental, and social health [8–12], and plays a pivotal role in shaping their overall quality of life [13]. Considering that, with increasing age, people spend more time in their immediate neighborhoods and become increasingly dependent on community relationships, resources, and services [14–16], unfavorable neighborhood conditions can be particularly detrimental to health in later life [17].

Research has shown that living in unsafe neighborhoods is associated with functional decline in older adults [18,19], as fear limits engagement in physical activity [20–22] and reduces confidence in one’s ability to be active [18,23]. Older adults who perceive their neighborhoods as unsafe report higher levels of depressive symptoms, chronic stress, anxiety disorders [24,25], and increased mortality [26,27], all of which are well-established risk factors for chronic conditions, owing to their negative impact on cardiovascular health, immune function, and sleep quality [28,29]. The adverse effects of perceived neighborhood safety on health remain significant even in longitudinal studies, underscoring their long-term impact on older adults [30].

Perceptions of neighborhood safety are often influenced by knowledge of or personal exposure to, crime [31]. The adverse impacts may be particularly severe for older persons who are victims of violent crimes, with crime victimhood negatively affecting their physical [32–34] and psychological health [35–39]. Research has consistently shown that both short- and long-term exposure to crime in one’s community affects health [7,32,33,40,41]. Crime victimhood can trigger mental health problems such as depression, PTSD, and anxiety [35–37], which are significant risk factors for chronic conditions, such as heart disease, diabetes, stroke, osteoporosis, and Alzheimer’s disease [42–44]. It can also lead to behavioral changes [45], including social withdrawal [46,47], reduced physical activity [20–22], and sleep disturbances [36,48], all of which are known risk factors for chronic conditions including heart diseases, obesity, and musculoskeletal disorders [49]. Psychological distress from crime victimhood can result in unhealthy coping mechanisms [36,50], including smoking, excessive alcohol consumption, and overeating, which further increase the risk of chronic conditions [49,51,52].

Associations between unsafe neighborhoods and multimorbidity have been observed globally. In the U.S, a study conducted in Tennessee found that higher levels of neighborhood crime were significantly associated with the spatial clustering of chronic illnesses [53]. A recent systematic scoping review of 72 studies across several countries, including the United States, the United Kingdom, Canada, Australia, and South Africa, found that unfavorable neighborhood traits, including neighborhood unsafety, were linked to higher rates of multimorbidity [54]. This review found that neighborhood insecurity contributes to multimorbidity through chronic stress, reduced access to health care, limited opportunities for physical activity, and other harmful environmental exposures [54]. Importantly, studies across both urban and rural settings have affirmed the role of neighborhood safety [9,55] and cohesion in shaping health globally [9,56].

Most studies investigating the relationship between neighborhood environments and chronic diseases have been conducted in developed countries. Research specifically targeting older adults in India is limited. Few studies have investigated the association between perceived neighborhood safety and diverse health outcomes, including sleep quality [57] and depression [43,58], among older Indians. Despite growing interest in the health implications of neighborhood conditions, few studies in India have examined these relationships in the context of chronic conditions and multimorbidity among older adults, especially focusing on both perceived safety and direct crime exposure.

As per the Crime in India – 2022 report published by the National Crime Records Bureau (NCRB), the overall crime rate in India declined slightly from 445.9 per 100,000 population in 2021 to 422.2 in 2022 [59,60]. That said, there was an increase in certain types of crime. Crimes against older adults, for instance, went up by 9% in 2022 compared with the previous year [59,60]. A recent study found that crimes such as theft, burglary, and fraud targeting older Indians have increased in large metropolitan cities such as Delhi, Mumbai, Chennai, Hyderabad, and Bengaluru [61]. Factors such as physical vulnerability, social isolation, cognitive decline, and economic challenges may contribute to older Indians’ higher susceptibility to crime [62,63]. The shift from joint family systems to nuclear families due to modernization and urbanization [64] is likely to exacerbate this issue, leading to increased social isolation and reduced familial support for older adults. With fewer extended family members available for support [64], present and future cohorts of older Indians may endure the risk of becoming increasingly isolated [65] and dependent on their immediate environments for social interaction and assistance.

The potential for increased reliance on one’s immediate surroundings highlights the importance of a safe and supportive environment. Given India’s rapidly aging population, high rates of violent crimes [66], and rising burden of chronic disease [2,67,68], this study aimed to examine the association between perceived neighborhood safety, crime victimization, and the prevalence of chronic conditions and multimorbidity among older adults in India.

We also considered the extent to which the associations between neighborhood contexts and chronic conditions, as well as multimorbidity, were moderated by functional disabilities. The press-competence model [69] posits that human behavior and function result from the interaction between an individual’s competencies and the environmental demands or “press.” As individual competence decreases with age, older adults are more susceptible to environmental effects [70]. Choi et al. (2018) applied this model and found that older adults who viewed their neighborhoods as unsafe reported poorer psychological health, with the effect especially pronounced among those with functional limitations [12]. Functional disabilities, defined as difficulties in ADLs and IADLs, can restrict mobility and physical activity [71], a restriction worsened by unsafe neighborhoods [20–22], leading to increased chronic conditions [28,29]. These limitations also heighten social isolation [72,73], exacerbated by perceived neighborhood unsafety [46,47], compounding mental health, and chronic disease issues. Access to healthcare may also become more challenging in unsafe areas [74], delaying treatment and management of chronic conditions. Additionally, functional limitations may impair older adults’ ability to respond to threats in unsafe environments [75], intensifying psychological and physical stress and further increasing the risk of chronic conditions. Overall, the combined stress of functional limitations and unsafe environment can increase the risk of developing chronic conditions. An additional objective of this study was to evaluate the degree to which the associations between neighborhood safety, crime victimization, chronic conditions, and multimorbidity were moderated by difficulties in ADLs and IADLs.

## Methods

### Data and sample

The data were drawn from the World Health Organization’s (WHO) Study on Global AGEing and Adult Health (SAGE), India wave-2, conducted in 2015. The data were accessed for research purposes on 1 September 2024 and at no point did the authors have access to personal identifiable information. This wave covered six diverse states of India, each representing a different region: Assam (Northeast), Karnataka (South), Maharashtra (West), Rajasthan (North), Uttar Pradesh (Central), and West Bengal (East). This study included a comprehensive sample of 9,116 respondents aged 18 years and above. SAGE Wave-2 in India was a follow-up to SAGE Wave-1, maintaining the same primary sampling units (PSUs) and households initially covered by the WHO World Health Survey (WHS) in 2003. In rural areas, a two-stage sampling process was employed, with villages as PSUs and households as secondary stage units (SSUs). In urban areas, a three-stage sampling process was utilized, involving the sequential selection of wards, census enumeration blocks, and households. The number of households selected was proportional to the respective state populations and was distributed across urban and rural areas. The Wave-2 study included 1,998 respondents aged 18–49 years and 7,118 older adults aged ≥ 50 years. In this study, we focused on 7,118 respondents aged ≥ 50 years. In the multivariable models, there were only 48 missing cases, which were handled using list-wise deletion.

### Ethics statement

This study involved secondary analysis of publicly available data from the World Health Organization’s SAGE Wave 2. The dataset was accessed directly from the International Institute for Population Sciences (IIPS), Mumbai on 20 April 2023. Ethical approval for primary data collection was obtained by the original investigators from the Institutional Review Board of IIPS, and informed consent was obtained from all participants at the time of data collection. As the dataset was de-identified and publicly accessible, no additional ethical approval was required for this secondary analysis. The authors did not have access to any personally identifiable information.

### Outcome variables

Chronic diseases refer to self-reported physician diagnoses of diseases such as hypertension, diabetes, stroke, arthritis, angina, asthma, and lung disease. The variable “number of chronic diseases” was created based on the presence of specific diseases. In this study, multimorbidity referred to the presence of two or more chronic diseases in older adults.

### Independent variables

Neighborhood safety was evaluated using two survey questions adopted from the World Bank’s Integrated Questionnaire for the Measurement of Social Capital [76]: [1] “In general, how safe do you feel from crime and violence when you are alone at home?” and [2] “How safe do you feel when walking down your street alone after dark?” (*r* = 0.65). These items have been used to assess neighborhood safety in various countries including India [43,77]. The responses were on a Likert scale (completely safe, very safe, moderately safe, slightly safe, and not safe at all). We reverse-coded the items and created a summary scale for neighborhood safety ranging from 0 to10, with a higher value reflecting a greater sense of neighborhood safety.

Crime exposure was measured by a single item, “In the last 12 months, have you or anyone in your household been the victim of a violent crime, such as assault or mugging?” The responses were yes or no.

### Moderators

Activities of daily living (ADLs) and instrumental activities of daily living (IADLs) were self-reported. In the SAGE survey, participants were asked to report difficulty in performing particular activities in the last 30 days on a five-point scale (none, mild, moderate, severe, and extreme). In this study, severe and extreme difficulties were combined. The ADLs included getting up from lying down, walking, standing for long, carrying things, eating, bathing, dressing, using the toilet, and transferring and concentrating for approximately 10 minutes (Cronbach’s alpha = 0.78). IADLs included using private/public transport, carrying out household responsibilities, joining community functions, doing day-to-day work, and getting out of the household (Cronbach’s alpha = 0.76). ADL and IADL difficulties were recoded as two dichotomous, yes or no variables and participants who reported severe or extreme difficulty in performing any ADL and IADL were coded as “yes” and otherwise “no.” For the sensitivity analysis, we categorized moderate, severe, and extreme difficulties as “yes,” and all other responses as “no”.

### Covariates

Based on the aforementioned studies, several sociodemographic and mental health-related variables were selected and included in the current analysis as control factors. The covariates included age, grouped into four categories: 50–59, 60–69, 70–79, and ≥ 80 years; sex, categorized as male or female; and educational level, classified as no education, less than primary, primary, secondary, and higher education. Current marital status was classified as married and unmarried (including widowed, never married, separated, or divorced). Additionally, given the impact of sleep and mental health on chronic conditions, we controlled sleep quality and the self-reported prevalence of depression among participants. Sleep quality was assessed using the question “Please rate the quality of your sleep last night. Was it very good, good, moderate, poor, or very poor?” Poor and very poor were recoded as poor and otherwise good. Prevalence of depression was assessed using the question, “Have you ever been diagnosed with depression?” with response as yes and no.

The household wealth index was calculated based on a detailed list of household assets and amenities, such as durable goods, housing characteristics (type of floors, walls, and cooking stoves), and access to improved water, sanitation, and cooking fuel. Principal component analysis was used to create the wealth index and divided it into five quintiles, with the lowest quintile representing the poorest households and the highest quintile representing the wealthiest households [78]. Religious groups included Hindus, Muslims, and others, while social groups comprised scheduled castes and scheduled tribes (both socioeconomically most disadvantaged) and others. The places of residence were classified as urban or rural, and the states included in the study were Assam, Karnataka, Maharashtra, Rajasthan, Uttar Pradesh, and West Bengal.

### Statistical analysis

In this study, descriptive statistics were reported to present the sample distribution and mean scores of the number of chronic conditions, along with standard deviation (SD) and percentage prevalence of multimorbidity across the explanatory variables. Linear and logistic regression models were used to fulfil the study objectives. In addition, we used interaction terms to examine the moderating role of ADL/IADL disabilities on the observed associations. In the multivariable analyses, we used different models to capture the effects of individual and household/community-related variables: Model 1 was adjusted for individual-level variables such as ADL/IADL difficulty, age, sex, education, marital status, sleep quality, and depression; model 2 was additionally adjusted for household and community-related variables such as household wealth quintile, religion, caste, place of residence, and states; and interaction models were adjusted for all the selected variables.

The results were presented as adjusted beta coefficients from linear regression models and odds ratios (OR) from logistic regression models, each with corresponding 95% confidence intervals (CIs). Individual weights were used to account for the sampling design and make the estimates nationally representative. Stata version 16 was used for all analyses [79]. The variance inflation factor (VIF) was calculated using Stata to assess multicollinearity among the selected variables, and no evidence of problematic multicollinearity was found, with all VIF values below 5 (see S1 Table). Survey weights were applied in all analyses to account for the complex survey design, enhancing the accuracy and reliability of the population-level estimates.

## Results

Table 1 presents the sample characteristics. A little more than half of the sample were females (52.39%), 5.14% were aged 80 + years, and 48.33% had no formal education. The mean score of neighborhood safety (on a scale of 0–10) was 7.72 (SD: 2.05). Approximately 6% of the participants reported that they or someone in their household were victims of a violent crime in the last one year. A total of 37.28% reported ADL difficulty, and 24.91% reported IADL difficulty.

**Table 1:** Background characteristics of study participants, SAGE- 2015 (n = 7,118).

Variables	n (%)/Mean (SD)
Neighborhood safety, Mean (SD)	7.72 (2.05)
Crime victimhood	
No	6684 (94.03)
Yes	434 (5.97)
Number of chronic conditions, Mean (SD)	0.61 (0.84)
Multimorbidity	
No	5953 (85.16)
Yes	1165 (14.84)
ADL difficulty	
No	4456 (62.72)
Yes	2662 (37.28)
IADL difficulty	
No	5385 (75.09)
Yes	1733 (24.91)
Age (in years)	
50-59	2904 (40.85)
60-69	2585 (36.02)
70-79	1285 (18)
80+	344 (5.14)
Sex	
Male	3337 (47.61)
Female	3781 (52.39)
Level of education	
No formal education	3574 (48.33)
Up to primary	1922 (26.68)
Secondary	675 (9.68)
Higher	947 (15.31)
Current marital status	
Married	5305 (74.87)
Unmarried	1813 (25.13)
Depression	
No	6948 (97.60)
Yes	170 (2.40)
Sleep quality	
Good	4,986 (71.50)
Moderate	1,791 (23.98)
Poor	295 (4.24)
Wealth quintile	
Poorest	1371 (20.13)
Poor	1304 (17.97)
Middle	1318 (18.06)
Rich	1468 (20.93)
Richest	1657 (22.90)
Religion	
Hindu	5966 (85.08)
Muslim	869 (11.91)
Others	279 (3.00)
Social group	
Scheduled castes	522 (6.32)
Scheduled tribes	1168 (14.68)
Other backward classes	3313 (49.56)
Others	2115 (29.44)
Place of residence	
Urban	1412 (28.48)
Rural	5606 (71.52)
States	
Assam	723 (5.06)
Karnataka	872 (11.21)
Maharashtra	1176 (21.14)
Rajasthan	1456 (12.2)
Uttar Pradesh	1534 (32.27)
West Bengal	1357 (18.13)

N: Unweighted counts; % Weighted percentages to account for sampling design; ADL: Activities of daily living; IADL: Instrumental ADL.

Table 2 presents the bivariate associations of neighborhood safety, crime exposure, ADL/IADL difficulty, and other background characteristics with the number of chronic conditions and multimorbidity. Overall, the mean number of chronic conditions among older adults was 0.66 (SD: 0.86), and 14.84% of the older adults had multimorbidity in this study. Neighbourhood safety was significantly negatively correlated with the number of chronic conditions (r = -0.09) and multimorbidity (r = -0.06). Older adults with crime exposure and ADL/IADL difficulties had a higher prevalence of chronic conditions and multimorbidity. Older adults in the 80 + years age group, those who were currently unmarried, those with higher levels of education, belonging to the higher wealth quintile, and residing in urban areas had a higher number of chronic conditions and multimorbidity. In addition, older adults with poor sleep quality and depression had higher numbers of chronic conditions and multimorbidity.

**Table 2:** Mean estimates of number of chronic conditions and prevalence of multimorbidity by neighborhood safety, crime victimhood and other background variables among older adults, SAGE- 2015.

Variables	Number of chronic conditions	Multimorbidity
	Mean (SD)	n (%)
Neighborhood safety^$^	***	***
Crime victimhood	**	*
No	0.66 (0.86)	1076 (14.71)
Yes	0.77 (0.88)	88 (17.68)
ADL difficulty	***	***
No	0.55 (0.79)	555 (11.41)
Yes	0.85 (0.94)	610 (20.60)
IADL difficulty	***	***
No	0.6 (0.82)	762 (12.60)
Yes	0.84 (0.95)	403 (21.59)
Age (in years)	***	***
50-59	0.56 (0.79)	373 (11.08)
60-69	0.71 (0.89)	472 (17.13)
70-79	0.75 (0.9)	246 (17.56)
80+	0.82 (0.95)	74 (19.11)
Sex	*	#
Male	0.64 (0.87)	516 (14.31)
Female	0.69 (0.86)	649 (15.32)
Level of education	***	***
No formal education	0.58 (0.8)	468 (11.10)
Up to primary	0.72 (0.91)	367 (18.32)
Secondary	0.8 (0.9)	135 (16.85)
Higher	0.76 (0.93)	195 (19.29)
Current marital status	**	**
Married	0.64 (0.85)	816 (14.21)
Unmarried	0.73 (0.89)	327 (16.90)
Depression	***	***
No	0.59 (0.83)	1,053 (13.98)
Yes	1.90 (0.78)	112 (64.32)
Sleep quality	***	***
Good	0.56 (0.82)	761 (13.40)
Moderate	0.69 (0.85)	320 (16.75)
Poor	1.03 (1.01)	79 (28.83)
Wealth quintile	***	***
Poorest	0.5 (0.77)	159 (10.11)
Poor	0.6 (0.83)	185 (11.75)
Middle	0.64 (0.86)	198 (13.36)
Rich	0.67 (0.82)	233 (14.60)
Richest	0.86 (0.95)	390 (22.80)
Religion	**	*
Hindu	0.65 (0.85)	949 (14.32)
Muslim	0.74 (0.92)	168 (17.47)
Others	0.76 (0.92)	48 (19.33)
Social group	**	**
Scheduled castes	0.52 (0.78)	61 (11.20)
Scheduled tribes	0.63 (0.84)	172 (12.84)
Other backward classes	0.67 (0.87)	556 (15.20)
Others	0.7 (0.87)	376 (16.01)
Place of residence	***	***
Urban	0.87 (0.95)	370 (19.83)
Rural	0.61 (0.83)	795 (12.85)
States	***	***
Assam	0.92 (0.94)	183 (26.08)
Karnataka	0.75 (0.89)	164 (19.97)
Maharashtra	0.66 (0.87)	190 (14.53)
Rajasthan	0.69 (0.85)	237 (17.18)
Uttar Pradesh	0.38 (0.69)	127 (7.58)
West Bengal	0.77 (0.9)	264 (20.23)
Total	0.66 (0.86)	1165 (14.84)

SD: Standard deviation; n: Unweighted counts; Means and percentages are weighted to account for sampling design; p-values for number of chronic conditions are based on ANOVA test and p-values for multimorbidity are based on Chi-square test; ^$^ corresponding p-values for neighborhood safety are based on Pearson correlations; *** p < 0.001, ** p < 0.01, * p < 0.05, # p < 0.10; ADL: Activities of daily living; IADL: Instrumental ADL.

Table 3 presents the multivariable linear and logistic regression estimates of the number of chronic conditions and multimorbidity among older adults based on neighborhood safety, crime exposure, and other background characteristics. After adjustment for socioeconomic, demographic, and mental health-related variables, older adults with improved neighborhood safety had a lower number of chronic conditions (beta: -0.03, CI: -0.04, -0.01) and lower odds of multimorbidity (OR: 0.95, CI: 0.91, 0.99). Older adults with crime exposure had a higher number of chronic conditions (beta: 0.10, CI: 0.02, 0.19) than those who did not. Moreover, older adults with higher education and those belonging to the highest household wealth quintile had a greater number of chronic conditions (higher education: beta: 0.19, 95% CI: 0.10, 0.28; richest: beta: 0.28, 95% CI: 0.20, 0.36) and higher odds of multimorbidity (higher education: OR: 2.11, 95% CI: 1.54, 2.89; richest: OR: 2.17, 95% CI: 1.59, 2.97) compared to those with no formal education and belonging to the poorest wealth quintile, respectively. Those residing in rural areas had a lower number of chronic conditions (beta: -0.07, 95% CI: -0.14, -0.01) and lower odds of multimorbidity (OR: 0.75, 95% CI: 0.60, 0.93) than their peers from urban areas. Likewise, those reporting depression and poor sleep quality had more chronic conditions (depression: beta: 1.16, 95% CI: 1.03, 1.29; poor sleep: beta: 0.28, 95% CI: 0.10, 0.45) and greater odds of multimorbidity (depression: OR: 9.91, 95% CI: 6.52, 15.08; poor sleep: OR: 1.73, 95% CI: 1.14, 2.63) than those without depression and those with good sleep quality.

**Table 3:** Regression estimates of number of chronic conditions and multimorbidity by background variables among older adults SAGE- 2015.

Variables	Number of chronic conditions (beta coefficients)		Multimorbidity (odds ratios)		Number of chronic conditions (beta coefficients)	Multimorbidity (odds ratios)
	Model 1	Model 2	Model 1	Model 2	Interaction models	
Neighborhood safety	-0.04*** (-0.05, -0.03)	-0.03*** (-0.04, -0.01)	0.92*** (0.89, 0.96)	0.95* (0.91, 0.99)		
Crime victimhood						
No	Ref.	Ref.	Ref.	Ref.		
Yes	0.08 (-0.00, 0.17)	0.10* (0.02, 0.19)	1.18 (0.88, 1.59)	1.23 (0.90, 1.68)		
ADL difficulty						
No	Ref.	Ref.	Ref.	Ref.		
Yes	0.19*** (0.13, 0.25)	0.22*** (0.16, 0.27)	1.65*** (1.35, 2.02)	1.89*** (1.54, 2.32)		
IADL difficulty						
No	Ref.	Ref.	Ref.	Ref.		
Yes	0.07* (0.00, 0.13)	0.12*** (0.06, 0.18)	1.31* (1.06, 1.62)	1.54*** (1.23, 1.92)		
Age (in years)						
50-59	Ref.	Ref.	Ref.	Ref.		
60-69	0.15*** (0.09, 0.21)	0.15*** (0.10, 0.21)	1.61*** (1.31, 1.99)	1.65*** (1.34, 2.04)		
70-79	0.17*** (0.09, 0.24)	0.16*** (0.09, 0.23)	1.57** (1.19, 2.06)	1.51** (1.14, 2.00)		
80+	0.18** (0.05, 0.30)	0.13* (0.01, 0.26)	1.64* (1.10, 2.43)	1.45 (0.96, 2.19)		
Sex						
Male	Ref.	Ref.	Ref.	Ref.		
Female	0.14*** (0.09, 0.20)	0.09** (0.03, 0.14)	1.57*** (1.27, 1.94)	1.32* (1.07, 1.62)		
Level of education						
No formal education	Ref.	Ref.	Ref.	Ref.		
Up to primary	0.22*** (0.16, 0.29)	0.14*** (0.08, 0.20)	2.37*** (1.89, 2.97)	1.93*** (1.55, 2.42)		
Secondary	0.31*** (0.23, 0.39)	0.19*** (0.11, 0.28)	2.47*** (1.81, 3.36)	1.76** (1.25, 2.46)		
Higher	0.35*** (0.26, 0.44)	0.19*** (0.10, 0.28)	3.31*** (2.50, 4.37)	2.11*** (1.54, 2.89)		
Current marital status						
Married	Ref.	Ref.	Ref.	Ref.		
Unmarried	-0.02 (-0.08, 0.04)	-0.03 (-0.08, 0.03)	1.04 (0.84, 1.28)	1.00 (0.81, 1.25)		
Depression						
No	Ref.	Ref.	Ref.	Ref.		
Yes	1.24*** (1.10, 1.38)	1.16*** (1.03, 1.29)	11.07*** (7.41, 16.54)	9.91*** (6.52, 15.08)		
Sleep quality						
Good	Ref.	Ref.	Ref.	Ref.		
Moderate	0.09*** (0.04, 0.15)	0.06* (0.01, 0.11)	1.21 (1.00, 1.46)	1.11 (0.91, 1.35)		
Poor	0.35*** (0.17, 0.53)	0.28** (0.10, 0.45)	2.01** (1.32, 3.05)	1.73** (1.14, 2.63)		
Wealth quintile						
Poorest		Ref.		Ref.		
Poor		0.01 (-0.05, 0.07)		0.99 (0.74, 1.33)		
Middle		0.01 (-0.05, 0.08)		1.08 (0.80, 1.47)		
Rich		0.10** (0.03, 0.17)		1.29 (0.94, 1.77)		
Richest		0.28*** (0.20, 0.36)		2.17*** (1.59, 2.97)		
Religion						
Hindu		Ref.		Ref.		
Muslim		0.08* (0.01, 0.16)		1.46** (1.13, 1.89)		
Others		0.11 (-0.03, 0.25)		1.09 (0.71, 1.68)		
Social group						
Scheduled castes		Ref.		Ref.		
Scheduled tribes		0.07 (-0.01, 0.16)		1.21 (0.82, 1.80)		
Other backward classes		0.13** (0.05, 0.21)		1.31 (0.91, 1.90)		
Others		0.10* (0.01, 0.18)		1.19 (0.81, 1.75)		
Place of residence						
Urban		Ref.		Ref.		
Rural		-0.07* (-0.14, -0.01)		0.75** (0.60, 0.93)		
States						
Assam		Ref.		Ref.		
Karnataka		-0.09 (-0.19, 0.01)		0.76 (0.56, 1.05)		
Maharashtra		-0.26*** (-0.35, -0.16)		0.53*** (0.38, 0.73)		
Rajasthan		-0.18*** (-0.27, -0.09)		0.64** (0.47, 0.86)		
Uttar Pradesh		-0.49*** (-0.58, -0.40)		0.24*** (0.17, 0.32)		
West Bengal		-0.11* (-0.20, -0.02)		0.67** (0.51, 0.89)		
Neighborhood safety X No ADL difficulty					-0.04*** (-0.05, -0.03)	0.89*** (0.85, 0.94)
Neighborhood safety X Yes ADL difficulty					-0.01 (-0.02, 0.00)	0.99 (0.95, 1.04)
Neighborhood safety X No IADL difficulty					-0.03*** (-0.05, -0.02)	0.92*** (0.88, 0.96)
Neighborhood safety X Yes IADL difficulty					-0.01 (-0.02, 0.01)	1.01 (0.97, 1.06)
No crime victimhood X No ADL difficulty					Ref.	Ref.
No crime victimhood X Yes ADL difficulty					0.26*** (0.21, 0.31)	2.17*** (1.80, 2.63)
Yes crime victimhood X No ADL difficulty					0.01 (-0.09, 0.10)	0.74 (0.46, 1.20)
Yes crime victimhood X Yes ADL difficulty					0.49*** (0.34, 0.64)	3.68*** (2.42, 5.61)
No crime victimhood X No IADL difficulty					Ref.	Ref.
No crime victimhood X Yes IADL difficulty					0.23*** (0.17, 0.29)	2.13*** (1.74, 2.61)
Yes crime victimhood X No IADL difficulty					0.04 (-0.05, 0.14)	1.08 (0.71, 1.64)
Yes crime victimhood X Yes IADL difficulty					0.51*** (0.34, 0.69)	3.52*** (2.15, 5.77)

Ref. Referent group; *** p < 0.001, ** p < 0.01, * p < 0.05; Model 1 is adjusted for all the individual-level variables such as ADL/IADL difficulty, age, sex, education, marital status, sleep quality and depression; Model 2 is additionally adjusted for household and community-related variables such as household wealth quintile, religion, caste, place of residence and states; Interaction models are adjusted for all the selected variables.

Table 3 presents the interaction between ADL/IADL difficulties and neighborhood safety, crime exposure on the number of chronic conditions, and multimorbidity among older adults. Those with safe neighborhoods but with ADL/IADL difficulties had higher levels of chronic conditions and multimorbidity compared to those without ADL/IADL difficulties. Older adults with crime exposure and ADL/IADL difficulties had higher levels of chronic conditions and multimorbidity than their peers with no ADL/IADL difficulties.

## Discussion

This study explored the associations between perceived neighborhood safety, crime exposure, prevalence of chronic conditions, and multimorbidity among community-dwelling older individuals in India, with a specific focus on the moderating role of functional disabilities measured by difficulties in ADLs and IADLs. Our findings indicate that older Indians who perceive their neighborhoods as unsafe report more chronic conditions and have higher odds of multimorbidity. These findings are consistent with studies from developed nations, where unsafe neighborhoods are linked to increased stress, reduced physical activity, and higher rates of chronic diseases [11,18,23,24,80,81].

Living in unsafe neighborhood can lead to chronic mental distress, which adversely affects cardiovascular and immune function [11,28,82,83]. Fear of crime can limit outdoor movements and physical activity [18,23], thereby increasing the risk of obesity and related chronic ailments [27]. While perceived neighborhood safety can stimulate social interaction and social engagement, perceived unsafety in one’s immediate surroundings can engender powerlessness, mistrust, limited social contact, and increased social isolation [84]. These social stressors, especially mistrust and isolation, can increase the risk of a range of chronic health issues including cardiovascular disease, some cancers, and cognitive decline [85–88]. In fact, stress experienced through social disconnection triggers the same areas in the brain that are triggered during physical pain [89,90], underscoring the profound health consequences of living in unsafe or isolating environments.

Our findings also reveal that crime victimhood is adversely associated with an increased number of chronic ailments and multimorbidity, consistent with prior research indicating that crime victimhood can lead to both immediate and long-term health consequences [7,33,40,41]. Exposure to crime can lead to mental health conditions such as depression, anxiety, and PTSD, which may, in turn, contribute to subsequent chronic conditions [36,43,42]. The psychological trauma from victimization may also translate into unhealthy coping mechanisms [36,50], further increasing the risk of chronic diseases [91]. Physiologically, the stress response to victimization, marked by increased levels of cortisol and other stress hormones [29,92,93], can impair immune function and contribute to the onset of chronic disease onset [28,94]. Moreover, victimhood can lead to social withdrawal and isolation [46], reducing social support, and worsening loneliness and depression, both of which are linked to adverse health outcomes [33]. These findings highlight the relevance of broader support systems for mitigating the health consequences of crime victimization among older adults who have experienced crime.

We also observed that the associations between perceived neighborhood safety, crime victimization, chronic conditions, and multimorbidity were stronger among older adults with ADL/IADL limitations. This finding, although based on cross-sectional data, reflects the press-competence model, which suggests that individuals with lower functional competence are more vulnerable to environmental stressors [12]. Older adults with functional limitations may lack the physical and cognitive resources to effectively cope with unsafe surroundings, heightening both psychological and physiological stress. These limitations can hinder their ability to respond to threats and navigate their environment safely, intensify feelings of vulnerability, and trigger stress-related biological processes that promote chronic diseases [82]. Limited mobility can also restrict physical activity, contributing to obesity and related illnesses such as diabetes and cardiovascular disease [95,96]. Moreover, greater dependence on others for daily tasks may engender helplessness and depression, both of which are linked to poor health outcomes [30].

In addition to neighborhood safety and crime exposure, we also observed meaningful associations between sociodemographic characteristics, chronic conditions, and multimorbidity. For instance, higher education levels were associated with more chronic conditions and higher odds of multimorbidity. Individuals who are more educated may have better and more stable access to healthcare and, in turn, be more likely to be formally diagnosed [97–99]. It is also possible that those with more education report health conditions more accurately because of their greater health literacy and awareness [100]. Likewise, older Indians in the higher wealth quintiles and urban dwellers reported more diagnosed chronic ailments and endured higher odds of multimorbidity. While this finding may reflect better access to medical professionals and formal diagnoses [101–103], it may also indicate growing health burdens among more urbanized and economically advantaged individuals, potentially due to adverse lifestyle factors [67,104]. Moreover, although not statistically significant in multivariable models, unmarried older Indians had slightly higher rates of chronic illness in bivariate analysis, suggesting that the absence of spousal support may play a role in shaping later-life health [105]. These findings underscore the relevance of social and structural locations to health in later life.

Our findings also showed that depression and poor sleep quality are associated with a higher number of chronic conditions and greater odds of multimorbidity. These associations may reflect underlying physiological mechanisms, such as inflammation and dysregulation of the hypothalamic-pituitary-adrenal (HPA) axis, both of which are implicated in sleep disturbances and mental health disorders [106,107]. Additionally, depression and poor sleep may reduce motivation for health-promoting behaviors [108,109], increase healthcare neglect [110], and increase vulnerability to stress-related illnesses [111], thereby exacerbating physical health conditions.

### Public health implications

This study’s findings have significant implications for public health. Although causal relationships cannot be inferred owing to the cross-sectional nature of the data, the observed associations point to several intervention opportunities. Increasing neighborhood safety through community policing, better street lighting, and neighborhood watch programs could improve both perceived and actual safety, thus benefiting older adults’ health. For those who have experienced a crime, access to psychological support and counselling may help reduce adverse effects.

Addressing the needs of older adults with ADL and IADL limitations through home modifications, assistive devices, and support services could help reduce their vulnerability to environmental stressors and improve their overall health. Public awareness campaigns highlighting the impact of crime on older adults’ mental and physical health could encourage greater community involvement in crime prevention initiatives. Finally, strengthening strong social networks within unsafe neighborhoods can build social cohesion, reduce social isolation, and support healthy aging.

### Strengths and limitations

To our knowledge, this is the first study to evaluate the link between perceived neighborhood safety, crime victimization, functional limitations, and chronic conditions among older adults in India, a context that is often underrepresented in the literature. The use of a large representative sample improves the generalizability of the findings, whereas considering functional limitations as a moderator provides a nuanced understanding of the interactions between individual capacities and environmental stressors. Moreover, the use of perceptions of neighborhood safety adds to the strengths of this study. Ample evidence links neighborhood environments with morbidity [112]. However, most inferences are based on objective traits measured with administrative data or researcher observations [12,112,113]. Studies using residents’ perceptions of their neighborhoods show inconsistent results, as perceptions are influenced by both observable and unobservable elements [10,12,114,115]. In the present study, we addressed how a perceived neighborhood context, namely, safety within one’s immediate surroundings, is linked to chronic health conditions among older Indians.

Alongside these strengths, we noted some important limitations of our study. *First*, the cross-sectional design of the study limits its ability to infer causality or even temporality. *Second*, reliance on self-reported data for chronic conditions may introduce reporting bias. Future studies should incorporate more objective health data. Similarly, although the subjective appraisal of neighborhood safety constitutes a strength of this study, studies replicating our research should consider using both subjective and objective measures of neighborhood safety. Subjective reports of crime victimization among older adults may also be affected by recall bias, perception inaccuracies, social desirability bias, and emotional or psychological factors, leading to over- or under-reporting. Types of crime may also play a role, an aspect that we were unable to consider with these data. *Third*, although we considered several conceptually relevant covariates, residual bias is always possible. In particular, we did not account for potential confounders, such as social support networks and living arrangements, both of which could influence the observed associations.

## Conclusion

Our study highlights the significant connections between perceived neighborhood safety, crime victimization, and the prevalence of chronic diseases and multimorbidity among older individuals in India, with these associations being particularly strong among those with ADL and IADL limitations. These findings suggest a need for far-reaching public health strategies that consider the environmental, social, and individual determinants of health to improve the health and wellness of older Indians. However, given the cross-sectional nature of our data, these associations should be interpreted with caution and further longitudinal research is required to establish causality.

## Supporting information

S1 Table Variance Inflation Factor (VIF) and 1/VIF (tolerance) values of the selected variables. (DOCX)
