# Supplementary material for: Perceived neighborhood safety, crime exposure, and chronic diseases among older Indians: The role of functional disabilities
Source: PLOS Glob Public Health. 2025 Sep 24;5(9):e0005151. doi: 10.1371/journal.pgph.0005151 (PMC12459815; doi:10.1371/journal.pgph.0005151)
Supplement: S1 Table — (DOCX) [file pgph.0005151.s001.docx]

| **S1 Table. Variance Inflation Factor (VIF) and 1/VIF (tolerance) values of the selected variables** | | |
| --- | --- | --- |
| Variables | VIF Values | 1/VIF (Tolerance Values) |
| Neighborhood safety | 1.21 | 0.82 |
| Crime victimhood |  |  |
| No | Ref. | Ref. |
| Yes | 1.02 | 0.98 |
| ADL difficulty |  |  |
| No | Ref. | Ref. |
| Yes | 1.54 | 0.65 |
| IADL difficulty |  |  |
| No | Ref. | Ref. |
| Yes | 1.15 | 0.87 |
| Age (in years) |  |  |
| 50-59 | Ref. | Ref. |
| 60-69 | 1.25 | 0.80 |
| 70-79 | 1.32 | 0.76 |
| 80+ | 1.2 | 0.83 |
| Sex |  |  |
| Male | Ref. | Ref. |
| Female | 1.44 | 0.70 |
| Level of education |  |  |
| No formal education | Ref. | Ref. |
| Upto primary | 1.42 | 0.70 |
| Secondary | 1.34 | 0.75 |
| Higher | 1.72 | 0.58 |
| Current marital status |  |  |
| Married | Ref. | Ref. |
| Unmarried | 1.26 | 0.79 |
| Depression |  |  |
| No | Ref. | Ref. |
| Yes | 1.02 | 0.98 |
| Sleep quality |  |  |
| Good | Ref. | Ref. |
| Moderate | 1.07 | 0.94 |
| Poor | 1.06 | 0.95 |
| Wealth quintile |  |  |
| Poorest | Ref. | Ref. |
| Poor | 1.61 | 0.62 |
| Middle | 1.69 | 0.59 |
| Rich | 1.78 | 0.56 |
| Richest | 2.11 | 0.47 |
| Religion |  |  |
| Hindu | Ref. | Ref. |
| Muslim | 1.11 | 0.90 |
| Others | 1.05 | 0.95 |
| Social group |  |  |
| Scheduled castes | Ref. | Ref. |
| Scheduled tribes | 2.87 | 0.35 |
| Other backward classes | 4.79 | 0.21 |
| Others | 4.36 | 0.23 |
| Place of residence |  |  |
| Urban | Ref. | Ref. |
| Rural | 1.21 | 0.83 |
| States |  |  |
| Assam | Ref. | Ref. |
| Karnataka | 3.11 | 0.32 |
| Maharashtra | 4.4 | 0.23 |
| Rajasthan | 3.35 | 0.30 |
| Uttar Pradesh | 4.67 | 0.21 |
| West Bengal | 4.07 | 0.25 |
| Mean VIF | 2.09 |  |
